# Matching degree evaluation between new urbanization and carbon emission system in China: a case study of Anhui Province in China

**DOI:** 10.1038/s41598-023-38971-4

**Published:** 2023-07-20

**Authors:** Gou Yanfeng, Xing Qinfeng, Yang Ziwei

**Affiliations:** State Key Laboratory of Mining Response and Disaster Prevention and Control in Deep Coal Mines, Anhui University of Science and Technology, Huainan, 232001 Anhui China

**Keywords:** Environmental sciences, Environmental social sciences, Risk factors

## Abstract

In order to reveal the relationship between new urbanization and carbon emission to provide reference opinions for the construction of low-carbon urbanization, an evaluation system between new urbanization and carbon emission was constructed. Then their matching degree relationship was analyzed by coupling coordination degree model based on the data from 2012 to 2021 in Anhui Province, and their development trend from 2023 to 2032 was predicted by gray prediction model. The results show that: (1) New urbanization and carbon emission have the co-trend effect, and the consistency of core impact factors is relatively significant. Among them, the level of new urbanization increases from 0.058 in 2012 to 0.699 in 2021 and carbon emission development increases from 0.023 in 2012 to 0.165 in 2021, which both showing an upward trend. Meanwhile, social urbanization and population carbon emission are the core influencing factors. (2) The coupling coordination degree between new urbanization and carbon emission is low, but the synergy trend is optimistic and there is a large room for improvement. Among them, the coupling coordination coefficient of the coupling system rises from 0.136 in 2012 to 1.412 in 2021 (antagonistic phase), and then reaches 0.820 by 2032 (highly coordinated phase) by forecast. It shows that their current development is unbalanced, but the development trend is good, and there is a chance for improvement. This paper deepens the understanding of the logical correlation between new urbanization and carbon emission, and the following views are formed: (1) Low-carbon development is still the mainstream of new urbanization; (2) The coordination development of new urbanization and carbon emission reduction should be strengthened.

## Introduction

The development of urbanization is the embodiment of industrial agglomeration effect and economies of scale, which also leads to the continuous increase of carbon emission^1–3^. It is in this context that the concept of new urbanization has been presented in China. The new urbanization is to fully integrate the concept and principle of ecological civilization into the whole process of urbanization, and take the road of low-carbon urbanization^4^. In order to reveal the coupling relationship between new urbanization and carbon emission, and then provide reference for the construction of low-carbon urbanization, related academic research have been greatly presented.

In the 1970s, the concept of "urbanization" began to be recognized in China^5–7^. Then, many scholars have put forward different indicator systems to measure the level of urbanization from different perspectives, such as population, economy, society, urban infrastructure and ecology^8–12^. As for carbon emission, there are usually three indicators to evaluate the level of carbon emission, such as total carbon emission, per capita emission and CO_2_ emission^13^. Moreover, population size, industrial structure, growth rates of economic, levels of de-industrialization and urbanization rate also have different effects on carbon emission^14,15^.

Furthermore, relevant studies on the relationship between new urbanization and carbon emission have emerged in an endless stream. For example, some scholars analyzed the Kuznets curve relationship between urbanization and carbon emission via gray correlation model and Kuznets curve model from three aspects of land, population and economic urbanization^16,17^. Other scholars chose the spatial Durbin model to analyze the impact of urbanization on carbon emission based on four dimensions of population, economic, consumption and residential urbanization^18,19^. In the current research phase, the mainstream views between new urbanization and carbon emission can be summarized as follows: (1) Urbanization will not only increase urban population density, but also increase the pressure on infrastructure construction. The combined effect of these two aspects is a direct source of the increase in carbon emission intensity and an important obstacle on the road to carbon emission reduction^16–21^. (2) Urbanization is conducive to the reduction of carbon emission, and the effect of carbon emission reduction is negatively correlated with the level of economic development^22–26^. However, the academic community rarely pays direct attention to the matching relationship between new urbanization and carbon emissions, and lacks scientific intervention for the core influencing factors.

Based on the above literature review, an evaluation system of new urbanization from the five sub-dimensions of population, economy, society, ecology and innovation, and an evaluation system of carbon emission from the three sub-dimensions of population, economy and energy are constructed to analyze their coupling relationship and development trend in Anhui province.

## Study design and data sources

### Study area

**Figure 1:**
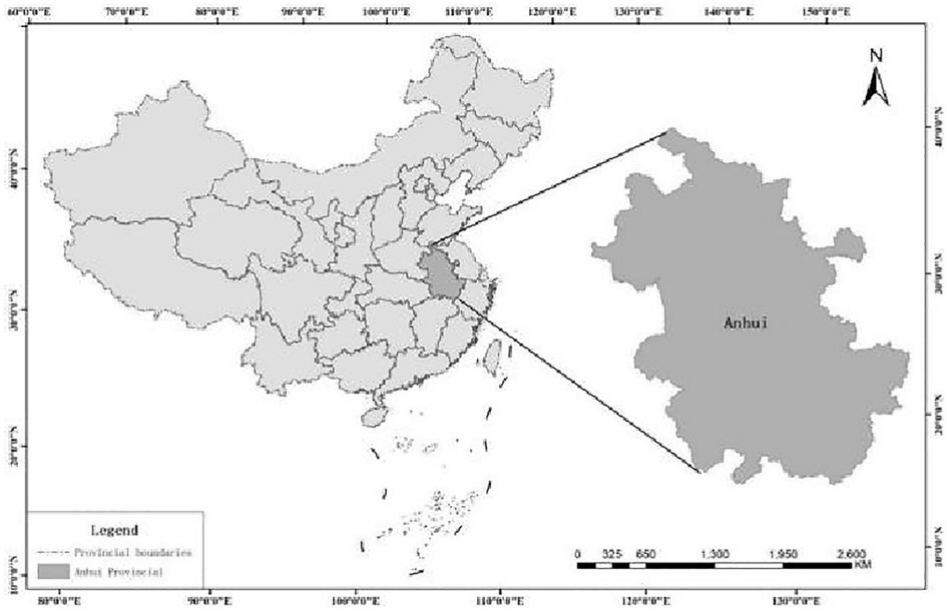
Location of Anhui Province in China. *Note* According to the location of Anhui Province in China, a map is formed through ARCGIS, and the specific location of Anhui Province has been marked on the right side of the figure.

The study area is shown in Fig. 1.

As an important part of the Yangtze River Delta city cluster, the economic development speed in Anhui Province is relatively backward and the urbanization has not reached the national average level, Especially to the Yangtze River Delta city cluster. Therefore, the new development concept throughout the whole process and all fields of new urbanization in Anhui Province began to be emphasized with the intensive and compact urban development and the low-carbon transformation as the lead. Furthermore, with the continuous advancement of new urbanization in Anhui Province, the interaction between new urbanization and carbon emission has attracted extensive attention from the society. So it is of practical research significance to take Anhui Province as the research object to analyze the coupling relationship between new urbanization and carbon emission, and then provide reference for the construction of low-carbon urbanization in Anhui Province and other provinces in China.

### Data sources and indicators

#### Data sources

The selected indicator data were obtained from the National Bureau of Statistics of China and the Statistical Yearbook of Anhui Province (2012–2021), and the missing data were obtained by interpolation. Interpolation is a widely used method for finding unknown data through the known by using equivalence in mathematical disciplines^27–29^. In order to make the data more relevant, this study used excel polynomial trend lines for the unknown data, which resulted in a better fit of the data trend lines^30^.

#### Indicators

Based on the basic principles of scientificity, rationality and usability, the new urbanization evaluation indicator system including population urbanization, economic urbanization, social urbanization, ecological urbanization and innovation urbanization was constructed with reference to the connotation of new urbanization in the National New Urbanization Plan (2021–2035) and existing academic research above^31–38^. Then, the carbon emission evaluation indicator system including population carbon emission, economic carbon emission and energy carbon emission was constructed^39–41^. The specific indicators are described as follows:

(i) Population urbanization, which reflects the population factor dimension of new urbanization. The specific indicators under this dimension include the urbanization of resident population, urban population density and registered unemployment rate of urban population^42^. The urbanization rate and population density are the visual representation of the urbanization aggregation trend. The registered unemployment rate of urban populations reflects whether urbanization has brought employment opportunities and livelihood security to residents.

(ii) Economic urbanization, which reflects the economic factor dimension of new urbanization. The specific indicators under this dimension include GDP per capita, the proportion of output value of secondary industry to GDP, the proportion of output value of tertiary industry to GDP, and the growth rate of fixed asset investment^43,44^. GDP per capita measures economic development. The proportion of secondary and tertiary industries represents the industrial structure, which is an important part of the socioeconomic system. The growth rate of fixed asset investment represents the speed dynamics of infrastructure project construction in the urbanization process.

(iii) Social urbanization, which reflects the social factor dimension of new urbanization. Specific indicators under this dimension include the number of health technicians per 10,000 people, the number of college students per 100,000 people, the number of private car ownership, the ratio of per capita disposable income of urban and rural residents, and the number of urban and rural residents with low insurance^8–46^. Obviously, the above indicators represent the development of social security and education in the process of new urbanization.

(iv) Ecological urbanization, which reflects the ecological factor dimension of new urbanization. Specific indicators under this dimension include the greening coverage rate of urban built-up areas, per capita park green space area, household waste disposal rate, and per capita water resources. It represents the level of municipal health and ecological environment in the process of new urbanization.

(v) Innovation urbanization, which reflects the innovation factor dimension of new urbanization. Specific indicators under this dimension include the number of patents granted for inventions and R&D personnel^49,50^. Indispensable to the promotion of new urbanization is the soft power such as innovation and invention.

(vi) Population carbon emission, which is the carbon emission embodied in the new urbanization process at the dimension of population factors. The specific indicators under this dimension include electricity consumption and new energy vehicle production^51,52^. With the development of urbanization, the living standard and consumption level of the population will rise significantly, which leads to a gradual increase in the total electricity consumption and automobile consumption of the population, which directly affects the level of carbon emission.

(vii) Economic carbon emission, which is the carbon emission embodied in the process of new urbanization. The specific indicators under this dimension include the output value of strategic emerging industries and energy consumption per unit of GDP^53–55^. Strategic emerging industries are the deep integration of emerging technologies and emerging industries. Energy consumption per unit of GDP reflects changes in economic structure and energy utilization efficiency. Combined with related literature^56,57^, both of them can be chosen as economic indicators to measure carbon emission level.

(viii) Energy carbon emission, which is the carbon emission embodied in the process of new urbanization. The specific indicators under this dimension include total energy production and total energy consumption^58,59^. Both energy production and consumption can be categorized as energy activities, which are important sources of greenhouse gas emission and are energy-based indicators of carbon emission level.

### Indicator system construction

According to the selection of indicators, the matching degree evaluation indicator system between new urbanization and carbon emission is formed in Anhui Province in Table 1.

**Table 1:** Coupling Coordination evaluation system between new urbanization and carbon emission in Anhui Province.

Sub-system	First-indicator	Second-indicator	Unit	Symbol	Trend
New urbanization (U1)	Population urbanization (B1)	The urbanization rate of permanent residents	%	X1	+
		Urban population density	Person/km^2^	X2	+
		Registered unemployment rate of urban population	%	X3	−
	Economic urbanization (B2)	GDP per capita	RMB	X4	+
		The proportion of the output of the secondary industry in GDP	%	X5	−
		The proportion of tertiary industry output in GDP	%	X6	+
		Fixed asset investment growth rate	%	X7	+
	Social urbanization (B3)	Number of health professionals per 10,000 people	Person	X8	+
		Number of college students per 100,000 population	Person	X9	+
		Private car ownership	million units	X10	+
		Per capita disposable income ratio of urban and rural residents	%	X11	−
		Number of urban and rural residents receiving subsistence allowances	person	X12	−
	Ecological urbanization (B4)	Green coverage of urban built-up areas	%	X13	+
		Per capita park green area	m^2^	X14	+
		Harmless disposal rate of household garbage	%	X15	+
		Water resources per capita	m^3^/person·year)	X16	+
	Innovating urbanization (B5)	Number of invention patents granted	piece	X17	+
		R&D personnel	million people	X18	+
Carbon emission (U2)	Population carbon emission (B6)	Electricity consumption	million kWh	X19	−
		New energy vehicle output	million units	X20	+
	Economic carbon emission (B7)	Output value of strategic emerging industries	%	X21	+
		Energy consumption per unit of GDP	Ton of standard coal/RMB	X22	−
	Energy carbon emission (B8)	Total energy production	million tons of standard coal	X23	−
		Total energy consumption	million tons of standard coal	X24	−

*Note* The indicator tends to be "+", indicating that the larger the index, the more conducive to development; The indicator tends to be "−", indicating that the larger the indicator, the more inhibited the development.

The comprehensive development evaluation model is used to evaluate the matching degree relationship between new urbanization and carbon emission in Anhui Province, and the specific steps are given as follows:

(1) The dimensionless treatment of different tendency indicators can be obtained by formulas (1)–(2).

Positive trend:

1 $$yij = \frac{xij - \min xij}{{\max xij - \min xij}}$$

Negative trend:

2 $$yij = \frac{\max xij - xij}{{\max xij - \min xij}}$$

where, $x_{ij}$ is the value of the $j$ indicator in the $\mathrm{i}$ year, and the values with different trends can be normalized to $y_{ij}$ by formulas (1)–(2) to facilitate the non-differential processing of subsequent values.

(2) The information entropy $ej$ and the weights of each indicator $\omega_{j}$ can be obtained by formulas (3)–(5).

3 $$ej = - \frac{1}{\ln m}\sum\limits_{i = 1}^{m} {fij\ln fij}$$

4 $$fij = \frac{yij + 1}{{\sum\limits_{i = 1}^{m} {\left( {yij + 1} \right)} }}$$

5 $$\omega_{j} = \frac{1 - ej}{{n - \sum\limits_{j = 1}^{n} {ej} }}$$

where, $y_{ij}$ is the standardized value of the $\mathrm{j}$ indicator, which is obtained by formulas (1)–(2) in year $\mathrm{i}$, and $\mathrm{m}$ is the time category, which is 10 in this study. The entropy $ej$ of each indicator can be obtained by formulas (3)–(4). Then, the weights $\omega_{j}$ of each indicator are obtained by formula (5) and correspond to the importance of each indicator in the evaluation system.

(3) The comprehensive development level between new urbanization and carbon emission can be obtained by formulas (6)–(8).

6 $$F\left( {x{}_{1}} \right) = \sum\limits_{{j_{1} = 1}}^{{n_{1} }} {a_{j1} \times X_{j1} }$$

7 $$F\left( {x_{2} } \right) = \sum\limits_{{j_{2} = 1}}^{{n_{2} }} {{\text{a}}_{{j_{2} }} \times X_{j2} }$$

8 $$T = \alpha F\left( {x_{1} } \right) + \beta F\left( {x_{2} } \right)$$

where, $F\left( {x_{1} } \right)$ and $F\left( {x_{2} } \right)$ represent new urbanization and carbon emission development indicator respectively. $a_{j1}$ and $a_{j2}$ represent the weight of the $\mathrm{j}$ indicator of new urbanization and carbon emission respectively; $X_{j1}$ and $X_{j2}$ are the standardized values of the $\mathrm{j}$ indicator of new urbanization and carbon emission respectively. $n_{1}$ and $n_{2}$ are the indicators of new urbanization and carbon emission respectively. Formula (6) represents the new urbanization development coefficient. Formula (7) represents the carbon emission development coefficient. Formula (8) represents the development coefficient of the comprehensive development level between the two systems. Based on the research results of previous scholars, it is concluded that new urbanization and carbon emission are equally important^60,61^, so $\alpha = \beta = 0.5$. $T$ is the comprehensive development coefficient.

### Coupling coordination degree model

The coupling coordination degree model is commonly used to measure the coordination development level of several systems^62^. It can not only reveal the interaction relationship between new urbanization and carbon emission, but also find the effective path to realize the coordinated development through their internal dynamic relationship.

#### Coupling degree

The coupling degree model is used to the interaction between new urbanization and carbon emission system, which is the basis of the coupling coordination degree model. The specific step is formula (9).

9 $$C = \left( {\frac{{F\left( {x_{1} } \right)F\left( {x_{2} } \right)}}{{F\left( {x_{1} } \right) + F\left( {x_{2} } \right)^{2} }}} \right)^{\frac{1}{2}}$$

where, $C(0 \le C \le 1)$ reflects the coupling degree between new urbanization and carbon emission, and the value of it is positively correlated with the coupling between the two systems.

#### Coupling coordination degree

Compared with the coupling degree model, the coupling coordination degree model has higher stability and wider application scope. For the time series of the studied area, all of them can be evaluated and compared quantitatively. The specific step is formula (10).

10 $$D = \sqrt {C \times T}$$

where, the value of $\mathrm{D}$ obtained by formula (10) is the coupling coordination degree, which can be used to evaluate the effect between new urbanization and carbon emission.

#### Matching coefficient

The matching coefficient is used to analyze the relative lag between the development level between new urbanization and carbon emission. Then the influence relationship between the two systems is analyzed by combined with formula (10). The specific step is formula (11).

11 $$U = \frac{{F\left( {x_{1} } \right)}}{{F\left( {x_{2} } \right)}}$$

where, the value of $\mathrm{U}$ obtained by formula (11) is the matching coefficient, which can be used to observe and analyze the relative lag trend between new urbanization and carbon emission.

### Gray prediction model

Grey prediction model is a method to predict from a small amount of incomplete information by establishing a mathematical model.It is an effective tool for dealing with small sample prediction problems. The principle is to generate data series with strong regularity through correlation analysis of the original data, and then the corresponding differential equation model was used to predict the development trend^63–67^. The main contribution of the gray prediction model in this study is to predict the coordination degree between new urbanization and carbon emission for 2023–2032 in Anhui Province. The aim is to provide suggestions for the construction of low-carbon urbanization by combining the prediction of the coupling coordination phase.The specific steps are as follows.

First, the original data of new urbanization and carbon emission are sequentially constructed ($X^{\left( 0 \right)} = \left\{ {x^{\left( 0 \right)}_{\left( 1 \right)} ,x^{\left( 0 \right)}_{\left( 2 \right)} , \ldots x^{\left( 0 \right)} \left( n \right)} \right\},X^{\left( 0 \right)} \left( k \right) \ge 0,k = 1,2, \ldots n$, and then $r$ times cumulative sequence is generated in formula (12).

12 $$x^{\left( r \right)} \left( k \right) = \sum\limits_{i = 1}^{k} {x^{{\left( {r - 1} \right)}} \left( i \right)} ,k = 1,2, \ldots ,n,r \ge 1$$

where, this is a group of irregular values through the accumulation of formula (12) to form a group of regular values.

Second, the adjacent values are obtained to generate the sequence,Then the gray differential equation model is constructed in formulas (13)–(14).

13 $$z^{\left( 1 \right)} \left( k \right) = \alpha x^{\left( 1 \right)} \left( k \right) + \left( {1 - \alpha } \right)x^{\left( 1 \right)} \left( {k - 1} \right),k = 1,2, \ldots ,n,\;\alpha = 0.5$$

14 $$d\left( k \right) + az^{\left( 1 \right)} \left( k \right) = b$$

where, $d\left( k \right)$ is the gray derivative, $a$ is the development coefficient, $z^{\left( 1 \right)} \left( k \right)$ is the whitening background value, and $b$ is the gray action.

Third, the data matrix is constructed to obtain the corresponding function of time. Then the cumulative generation prediction sequence is obtained through the cumulative generation in formulas (15)–(17).

15 $$U = \left( {\begin{array}{*{20}c} a \\ b \\ \end{array} } \right),\;B = \left( {\begin{array}{*{20}c} { - z^{\left( 1 \right)} \left( 2 \right)} & 1 \\ { - z^{\left( 1 \right)} \left( 3 \right)} & 1 \\ \vdots & \vdots \\ { - z^{\left( 1 \right)} \left( n \right)} & 1 \\ \end{array} } \right),\;Y = \left( {\begin{array}{*{20}c} {x^{\left( 0 \right)} \left( 2 \right)} \\ {x^{\left( 0 \right)} \left( 3 \right)} \\ \vdots \\ {x^{\left( 0 \right)} \left( n \right)} \\ \end{array} } \right),\;Y = BU$$

16 $$x^{\left( 1 \right)} \left( k \right) = \left( {x^{\left( 0 \right)} \left( 1 \right) - \frac{b}{a}} \right)e^{{ - a\left( {k - 1} \right)}} + \frac{b}{a},k = 1,2, \ldots ,n$$

17 $$x^{\left( 0 \right)} \left( k \right) = x^{\left( 1 \right)} \left( k \right) - x^{\left( 1 \right)} \left( {k - 1} \right),k = 1,2, \ldots ,n$$

where, formulas (13) and (14) are the premises of formula (15), the gray prediction model can be expressed as $Y = BU$. The cumulative predicted value sequence $x^{\left( 1 \right)} \left( k \right)$ obtained from formula (16) can be used to get annual predicted value through the inverse process of formula (12), that is, the application of formula (17).

Finally, the predicted value is compared with the actual value to verify whether it passes the residual test in formula (18).

18 $$\varepsilon \left( k \right) = \frac{{x^{\left( 0 \right)} \left( k \right) - \hat{x}^{\left( 0 \right)} \left( k \right)}}{{x^{\left( 0 \right)} \left( k \right)}},k = 1,2, \ldots ,n$$

where, $\left| {\varepsilon \left( k \right)} \right| < 0.1$ indicates high residual test accuracy and prediction accuracy.

The prediction data verified by formula (18) has scientific and reasonable application value.

## Result analysis

### Influencing factors analysis of new urbanization and carbon emission system

According to formulas (1)–(2), the standardized values of the evaluation system during 2012–2021 can be obtained in Table 2.

**Table 2:** Standardized data of new urbanization and carbon emission in Anhui Province from 2012 to 2021.

Indicator	2021	2020	2019	2018	2017	2016	2015	2014	2013	2012
X1	0.1906	0.1750	0.1560	0.1359	0.1161	0.0920	0.0680	0.0437	0.0227	0
X2	0.2069	0.1749	0.1797	0.1418	0.1040	0.0757	0.0585	0.0337	0.0000	0.0248
X3	0.1813	0.1316	0.1608	0.1316	0.1170	0.0731	0.0877	0.0731	0.0439	0
X4	0.2232	0.1786	0.1682	0.1429	0.1036	0.0732	0.0507	0.0388	0.0209	0
X5	0.1561	0.1727	0.1628	0.1495	0.1279	0.1101	0.0819	0.0274	0.0117	0
X6	0.1606	0.1657	0.1644	0.1555	0.1275	0.1041	0.0760	0.0314	0.0147	0
X7	0.0671	0	0.0640	0.1045	0.0234	0.0718	0.0718	0.1513	0.2059	0.2402
X8	0.2900	0.2038	0.1315	0.1052	0.0855	0.0657	0.0592	0.0460	0	0.0131
X9	0.3227	0.2198	0.1265	0.0527	0.0545	0.0578	0.0761	0.0527	0.0373	0
X10	0.1327	0.1430	0.1534	0.1321	0.1723	0.0899	0.0621	0.0725	0.0422	0
X11	0.1815	0.1626	0.1260	0.1129	0.0995	0.0950	0.0941	0.0848	0.0435	0
X12	0.1620	0.1447	0.1471	0.1313	0.1601	0.1563	0.0631	0.0286	0.0068	0
X13	0.0984	0.1254	0.1529	0.1450	0.1333	0.1137	0.0941	0.0941	0.0431	0
X14	0.1566	0.1525	0.1484	0.1417	0.1236	0.1082	0.0747	0.0659	0.0283	0
X15	0.1144	0.1144	0.1144	0.1144	0.1131	0.1131	0.1093	0.1080	0.0990	0
X16	0.4728	0.1363	0	0.0522	0.0448	0.1275	0.0704	0.0474	0.0135	0.0351
X17	0.3532	0.1505	0.1120	0.1157	0.0997	0.0800	0.0475	0.0306	0.0107	0
X18	0.2219	0.1953	0.1499	0.0979	0.0886	0.0765	0.0687	0.0643	0.0371	0
X19	0	0.0449	0.0648	0.0908	0.1243	0.1441	0.1683	0.1769	0.1859	0
X20	0.3292	0.1359	0.1504	0.1517	0.0755	0.0626	0.0446	0.0334	0.0167	0
X21	0.2688	0.1926	0.1522	0.1232	0.0961	0.0664	0.0469	0.0369	0.0169	0
X22	0.1746	0.1643	0.1656	0.1328	0.1183	0.0934	0.0778	0.0606	0.0126	0
X23	0.1033	0.1445	0.1775	0.1314	0.1260	0.1122	0.0661	0.0933	0.0457	0
X24	0	0.0327	0.0657	0.0947	0.1078	0.1247	0.1433	0.1386	0.1547	0.1379

In Table 2, the original data of each indicator (X1-X24) from 2012 to 2021 were standardized to eliminate the dimensional differences. Then subsequent entropy and weight calculations can be carried out.

**Figure 2:**
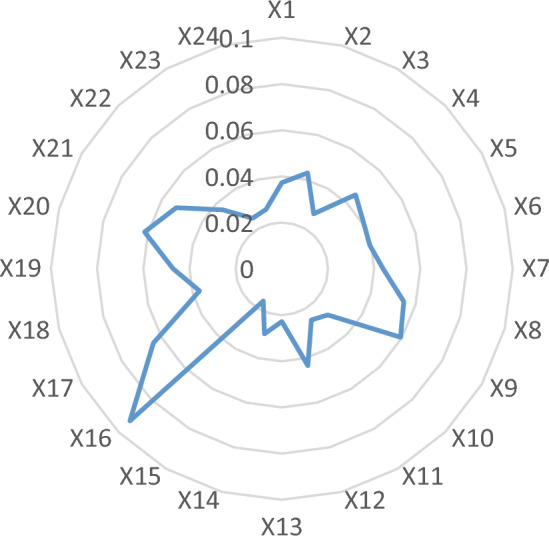
Weight distribution of each indicator of new urbanization and carbon emission system in Anhui Province.

According to formulas (3)–(5), the weights of each indicator between new urbanization and carbon emission in Anhui Province were obtained. Generally, the smaller the information entropy of an indicator is, the greater the variation degree of the indicator value will be and the more information will be provided. Its role in the comprehensive evaluation is positively correlated with the weight. The weights distribution of indicator in the evaluation system were shown in Fig. 2.

According to the basic principle of entropy method, it is intuitively found in Fig. 2 that X16 (per capita water resources) has the greatest influence in the new urbanization evaluation system (the weight is 0.093). The reason is that with the in-depth development of new urbanization, the growing demand for water resources result in the contradiction between supply and demand of water resources. Then the shortage of water resources in turn restrict the sustainable development of new urbanization. So the efficient use and effective protection of water resources should be focused on in Anhui Province.

Besides, the indicator of X20 (production of new energy vehicles) is the most important factor in the carbon emission evaluation system (the weight is 0.061). The figure reveals that the transformation of new energy vehicles have a crucial function on the energy conservation and emission reduction of the automobile industry. Meanwhile, the increase of the proportion of green electricity in the energy structure is an important reason for reducing carbon emission in Anhui province. Therefore, water conservation and energy structure adjustment should be regarded as the key link while taking into account other relevant aspects.

### Time series analysis between new urbanization and carbon emission system

#### Temporal evolution analysis of new urbanization level

**Figure 3:**
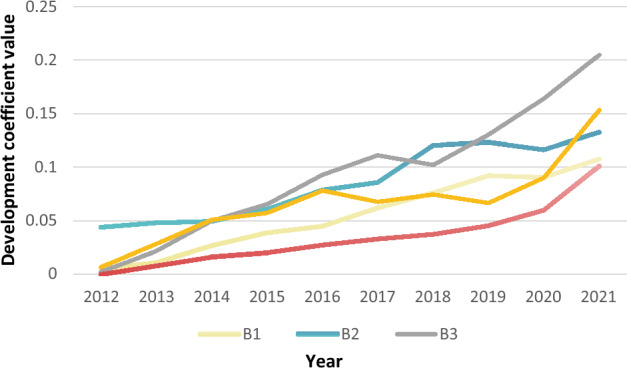
Temporal evolution analysis of new urbanization level in Anhui Province.

According to formulas (6)–(8) and the weight of each indicator in Fig. 2, the evolution trend of new urbanization level in Anhui Province from 2012 to 2021 is obtained in Fig. 3.

According to Fig. 3, the five sub-dimensions of new urbanization show an upward trend from 2012 to 2021. In combination with the actual situation, a plan for new urbanization under the policy guidance had been issued in Anhui Province. Among which, it emphasized that the concept of ecological civilization should be fully integrated into the development of new urbanization and promote the construction of low-carbon cities. This is reflected in the rapid growth of the five sub-dimensions of new urbanization in the development coefficient in 2014. In detail, B5 (innovation sub-dimension) has a stable upward trend, rising from 0.000 in 2012 to 0.101 in 2021. From B1 to B4 (population, economy, society and ecology sub-dimension respectively) show a zigzagging upward trend (B1 increased from 0.005 in 2012 to 0.107 in 2021; B2 increased from 0.044 in 2012 to 0.133 in 2021; B3 increased from 0.002 in 2012 to 0.205 in 2021; B4 increased from 0.007 in 2012 to 0.153 in 2021). Therefore, in the construction process of new urbanization, social resources, ecological environment, economic development and innovation capacity are all in the phase of continuous running-in. Additionally, the proportion of social and ecological sub-dimensions has gradually increased, forming a new urbanization development model dominated by society and ecology.

#### Temporal evolution analysis of carbon emission level

**Figure 4:**
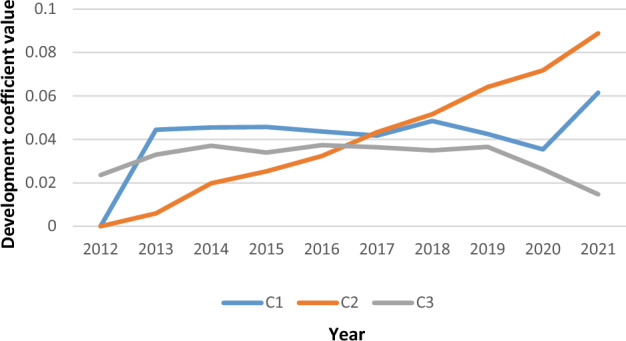
Temporal evolution analysis of carbon emission level in Anhui Province.

According to formulas (6)–(8) and the weight of each indicator in Fig. 2, the evolution trend of the carbon emission level in Anhui Province from 2012 to 2021 is obtained in Fig. 4.

According to Fig. 4, the three sub-dimensions of carbon emission present a significant heterogeneity trend. Specifically, from 2012 to 2021, C2 (economic carbon emission) development coefficient value increased to 0.089, the rising trend is stable. C3 (energy carbon emission) had been fluctuating around the value of 0.03 from 2012 to 2019, until 2021, showing a downward trend. C1 (population carbon emission) development coefficient value increased to 0.062, showing a zigzag upward trend. Overall, economic and population factors have a great impact on the level of carbon emission. Throughout the country, the economic strength in Anhui Province is in the middle and lower reaches, so more attention were paid to economic growth and less attention were paid to environmental issues. Additionally, with the emphasis on new urbanization, population carbon emission had decreased to a certain extent before 2020, but it is still at a high level. Meanwhile, population carbon emission had a trend of rising in 2021, which may be due to the proposal of Anhui Province's new urbanization plan (2021–2035). It requires efforts to improve the attractiveness and carrying capacity of cities, resulting in a turning increase in the level of carbon emission of the population. Besides, after experiencing a small fluctuation decline from 2012 to 2019, energy carbon emission showed a significant downward trend in the following two years, indicating that it had made great efforts to adjust the energy structure. In general, the level of carbon emission from population, economy and energy in Anhui Province is still fluctuating, so the carbon emission reduction is still facing severe challenges.

#### Comparative analysis of the comprehensive temporal evolution between new urbanization and carbon emission level

**Figure 5:**
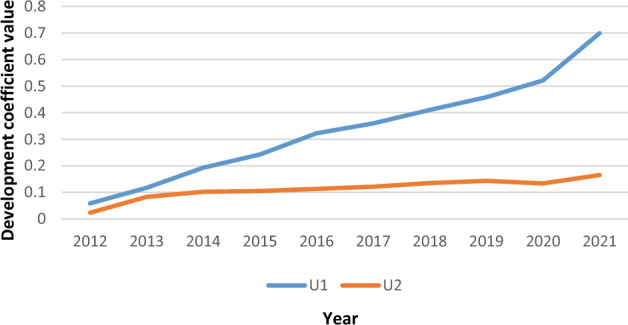
Comprehensive development coefficient of carbon emission level and new urbanization level in Anhui Province from 2012 to 2021.

The development coefficients of the five sub-dimensions under the new urbanization system in Fig. 3 and the three sub-dimensions under the carbon emission system in Fig. 4 are accumulated respectively. Then the evaluation trend of the comprehensive development coefficient between new urbanization and carbon emission is obtained in Fig. 5.

According to Fig. 5, the new urbanization coefficient rose from 0.058 to 0.699 from 2012 to 2021, indicating that the sustainable development ability in new urbanization has been significantly enhanced in Anhui Province. The main reason is that the Chinese government tried to implement the National New Urbanization Plan (2014–2020) and carried out a series of comprehensive pilot projects nationwide. Therefore, important progress has been made in the process of new urbanization. But the expansion of industries in the region to absorb population also leads to extremely prominent environmental pollution problems, and the carbon emission level is still rising slowly (the carbon emission level rose from 0.024 in 2012 to 0.165 in 2021). While the situation is still grim, the growth rate of carbon emissions is gradually slowing down because of the growing demand of urban residents for quality public services, ecological environment, health and safety. This phenomenon shows that there is making corresponding efforts for the process of carbon reduction in Anhui Province.

### Evaluation analysis of coupling coordination degree

In formulas (8)–(11), the comprehensive development coefficient (T), the coupling coefficient (C), the coupling coordination degree coefficient (D), the matching degree coefficient (U) of new urbanization and carbon emission system in Anhui Province during 2012–2021 are obtained respectively. The specific is shown in Table 3.

**Table 3:** Coupling coordination degree between new urbanization and carbon emission system in Anhui Province from 2012 to 2021.

Year	C	T	D	U
2012	0.453	0.041	0.136	2.475
2013	0.493	0.100	0.222	1.409
2014	0.476	0.148	0.265	1.887
2015	0.459	0.174	0.282	2.308
2016	0.439	0.218	0.309	2.840
2017	0.434	0.241	0.323	2.962
2018	0.432	0.273	0.343	3.038
2019	0.426	0.300	0.358	3.198
2020	0.403	0.327	0.363	3.906
2021	0.393	0.432	0.412	4.236

In Table 3, the coupling coordination degree between new urbanization and carbon emission system in Anhui Province had been on the rise during the studied period. The matching coefficient showed a trend of decreasing first and then increasing, but the development level of new urbanization is always better than the level of carbon emission. In general, the coupling degree of new urbanization and carbon emission is always in the antagonistic phase, and the coupling coordination degree has not reached the coupling coordination phase. Besides, the level of new urbanization is higher than the level of carbon emission. The reason is that with a series of major decisions and plans on the construction of new urbanization, the layout and form of urbanization have been further optimized. Meanwhile, the trend of sustainable urban development continues to rise, promoting the healthy development of new urbanization. However, the increase in urban population can promote economic development to a certain extent, but the increase in energy consumption and residential buildings will lead to an increase in carbon emissions, which will have negative impact on the urban ecological environment.

### Analysis of gray prediction model

Based on the principle of gray prediction model and formulas (12)–(17), the coupling coordination degree between new urbanization and carbon emission system in Anhui Province from 2012 to 2021 is predicted, and the actual value is compared with it. Then the relevant values are obtained in Table 4.

**Table 4:** Comparison of the predicted and actual values of the coupling coordination level between new urbanization and carbon emission system in Anhui Province from 2012 to 2021.

Year	Actual value	Predicted value	Residual
2012	0.136	0.136	0.000
2013	0.222	0.245	− 0.100
2014	0.265	0.261	0.017
2015	0.282	0.278	0.016
2016	0.309	0.296	0.042
2017	0.323	0.316	0.024
2018	0.343	0.336	0.020
2019	0.358	0.358	− 0.002
2020	0.363	0.382	− 0.052
2021	0.412	0.407	0.012

In Table 4, the residual value $\left| {\varepsilon \left( k \right)} \right| < 0.1$, and the accuracy of the prediction data is high, which verifies that the gray prediction model is suitable for this study. Then, the coupling coordination degree between new urbanization and carbon emission system in Anhui province from 2023 to 2032 is predicted. The specific predicted value of coupling coordination degree is shown in Table 5.

**Table 5:** Prediction of coupling coordination degree between new urbanization and carbon emission system in Anhui Province from 2023 to 2032.

Year	Predicted coupling coordination degree
2023	0.462
2024	0.493
2025	0.525
2026	0.560
2027	0.596
2028	0.636
2029	0.677
2030	0.722
2031	0.769
2032	0.820

In Table 5, the coupling coordination degree between new urbanization and carbon emission system in Anhui Province from 2023 to 2032 increases steadily from 0.462 to 0.820, indicating that the coupling coordination degree will not reach a highly coordinated phase until 2032. The reason is that the increase of urban population will not only promote economic growth, but also increase energy consumption, urban land use and other activities, resulting in a large amount of carbon emission^68–70^. The negative effects of new urbanization occupy a dominant position in the coupling system mechanism. But with the progress of time and the implementation of various relevant policies and measures, carbon emission issues in the process of new urbanization will be dealt with in an orderly manner, and the coordination degree between new urbanization and carbon emission coupling system will reach a highly coordinated phase in 2032. The negative impact of new urbanization on carbon emission will be gradually reduced^71,72^. New urbanization and low-carbon development gradually form a positive feedback relationship, and occupy a dominant position in the coupling system mechanism. Furthermore, in order to shorten the time for the coupling system to reach a highly coordinated phase, efforts should be made from the aspects of innovating low-carbon key technology, increasing financial support, strengthening social support and forming a real low-carbon production and life style.

## Discussion

This study takes Anhui Province as the research object, and constructs the comprehensive evaluation system between new urbanization and carbon emission. Later, the coupling coordination degree from 2012 to 2021 is analyzed in Anhui Province, and its development trend from 2023 to 2032 is predicted. Then the following views are formed.

### New urbanization and carbon emission have the co-trend effect, and the consistency of core impact factor is relatively significant

Combining the entropy weight method and the comprehensive development coefficient model, the development coefficient of new urbanization shows an overall upward trend (from 0.058 in 2012 to 0.699 in 2021), which is consistent with the result that Anhui Province is in the phase of accelerating urbanization development^73^. it is indicated that the five sub-dimensions have a significant promoting effect on new urbanization development. Furthermore, it is found that among the five sub-dimensions of new urbanization, social urbanization plays the most important role (the weight is 0.211).

As for carbon emission, its development coefficient generally showed a slow upward trend (from 0.024 in 2012 to 0.165 in 2021). Among the three sub-dimensions of carbon emission, population carbon emission plays the largest role (the weight is 0.109). Otherwise, except energy carbon emission, population carbon emission and economic carbon emission all showed an upward trend, and the situation of carbon emission reduction was still grim, so the focus of future carbon reduction should be on population and economic factors^74,75^.

### The coordination degree between new urbanization and carbon emission is low, but the synergy trend is optimistic and there is a large room for improvement

Combined with the coupled coordination model, the coupling coordination degree between new urbanization and carbon emission in Anhui Province from 2012 to 2021 is studied, and it is found that it has not broken through the confrontation phase to the coordination phase (the coupling coordination degree in 2021 is 0.412). However, through the gray prediction model, it is found that the development trend is optimistic and the room for improvement is large (the coupling coordination degree is predicted to be 0.820 by 2032, which is in the highly coordinated phase).

In the past, economic development and rapid urbanization led to increased consumption of natural resources, resulting in large amounts of carbon emission^76,77^, the negative effects of new urbanization dominate the coupling system. However, under the leadership of the Chinese government, Anhui Province adheres to the path of sustainable development, which forms a positive feedback mechanism. It is shown that the coupling system between new urbanization and carbon emission has great room for improvement. In order to shorten the time to reach a highly coordinated phase, the joint guidance of relevant policies and residents' low-carbon autonomy is needed^78,79^.

### Research contributions and limitations

The empirical results of this study can provide guidance for other provinces such as Anhui to realize the coordinated development between new urbanization and carbon emissions. Specifically, the hindrance factors between new urbanization and carbon emission development system should be identified, then the scientific suggestions can be presented to solve the corresponding problems in a targeted manner.

However, this study has some limitations, which need to be improved in future studies. First, a province in China is selected as the research object, which should be expanded to urban agglomeration and mega-cities in the future research. Second, the key indicators of the evaluation system are determined based on the research results of previous scholars. Therefore, in the further study, the selection of indicators should be investigated and innovated. Finally, in the selection of research methods, the method with higher applicability and scientific validity have been chosen, so different methods should be used for comparative analysis to get more scientific and appropriate research results.

## Conclusion

In order to promote the construction of new urbanization, and reduce the level of carbon emission, the following conclusions can be formed.

### The low-carbon development is still the mainstream of new urbanization construction

First, in the context of increasing pressure on the carbon emission, the bottom line of resource conservation should be firmly held, and the construction of low-carbon urbanization should be promoted. Second, the people-centered new urbanization must be kept in mind. Social urbanization for residents should be continuously improved, education and skills training for migrants should be continued, and urban governments should speed up implementation of their rights and interests protection. Finally, the double upgrading of urban hardware development quality and governance capacity modernization level should be promoted. The construction of a ecological urban should be accelerated to ensure the quality of new urbanization.

### The coordinated development of new urbanization and carbon reduction should be strengthened

First, the adjustment of the energy structure should be steadily advanced. In the context of China's active promotion of energy revolution, the development and utilization of non-fossil energy and the industrial development and technological research and development in the field of new energy should be promoted to the construction of new urbanization. Second, the development of low-carbon industry should be intensified. It is necessary to create more jobs for the people and provide new green drivers for the new urbanization. Finally, it is particularly necessary to advocate low-carbon lifestyle and let the concept of low-carbon life run through all aspects of the new urbanization.

## Data Availability

All data generated or analyzed during this study are included in this article.
